# Bovine Lactoferrin-Induced CCL1 Expression Involves Distinct Receptors in Monocyte-Derived Dendritic Cells and Their Monocyte Precursors

**DOI:** 10.3390/toxins7124897

**Published:** 2015-12-17

**Authors:** Daniela Latorre, Nadia Pulvirenti, Daniela Angela Covino, Barbara Varano, Cristina Purificato, Gabriella Rainaldi, Maria Cristina Gauzzi, Laura Fantuzzi, Lucia Conti, Gloria Donninelli, Manuela Del Cornò, Michela Sabbatucci, Sandra Gessani, Patrizia Puddu

**Affiliations:** Istituto Superiore di Sanità, Department of Hematology, Oncology and Molecular Medicine, Viale Regina Elena 299, Rome 00161, Italy; daniela.latorre@irb.usi.ch (D.L.); nadia.pulvirenti@gmail.com (N.P.); daniela.covino@iss.it (D.A.C.); barbara.varano@iss.it (B.V.); cristina.purificato@iss.it (C.P.); gabriella.rainaldi@iss.it (G.R.); mariacristina.gauzzi@iss.it (M.C.G.); laura.fantuzzi@iss.it (L.F.); lucia.conti@iss.it (L.C.); gloria.donninnelli@iss.it (G.D.); manuela.delcorno@iss.it (M.D.C.); michela.sabbatucci@iss.it (M.S.)

**Keywords:** monocyte, dendritic cell, lactoferrin, CCL1, surface receptors

## Abstract

Lactoferrin (LF) exhibits a wide range of immunomodulatory activities including modulation of cytokine and chemokine secretion. In this study, we demonstrate that bovine LF (bLF) up-modulates, in a concentration- and time-dependent manner, CCL1 secretion in monocytes (Mo) at the early stage of differentiation toward dendritic cells (DCs), and in fully differentiated immature Mo-derived DCs (MoDCs). In both cell types, up-modulation of CCL1 secretion is an early event following bLF-mediated enhanced accumulation of CCL1 transcripts. Notably, bLF-mediated up-regulation of CCL1 involves the engagement of distinct surface receptors in MoDCs and their Mo precursors. We show that bLF-mediated engagement of CD36 contributes to CCL1 induction in differentiating Mo. Conversely, toll-like receptor (TLR)2 blocking markedly reduces bLF-induced CCL1 production in MoDCs. These findings add further evidence for cell-specific differential responses elicited by bLF through the engagement of distinct TLRs and surface receptors. Furthermore, the different responses observed at early and late stages of Mo differentiation towards DCs may be relevant in mediating bLF effects in specific body districts, where these cell types may be differently represented in physiopathological conditions.

## 1. Introduction

Lactoferrin (LF) is an iron binding glycoprotein belonging to the transferrin family that is considered a major component of the mammal’s innate immune system. LF is found primarily in mucosal secretions, synthesized by epithelial cells, but also stored in specific neutrophilic granules [1]. LF is a first-line defense protein involved in protection against a multitude of microbial infections and prevention of systemic inflammation, thus helping the host to fight against microbes but also protecting it against the harmful effects of inflammation [2,3,4]. Although the role of LF in maintaining immune system homeostasis has not been fully elucidated, some regulatory activity may depend on its interaction with pathogen-associated molecular patterns and other surface receptors expressed on innate cells including monocytes (Mo), macrophages and dendritic cells (DCs) [5,6].

The mononuclear phagocyte system is a body-wide network of specialized cells such as macrophages and DCs, that play distinct and complementary immunological roles and overall contribute to tissue homeostasis, inflammation, and immune defense [7]. Mo continuously migrate from the blood into peripheral tissues where, in response to environmental stimuli, they differentiate into DCs [8]. These latter cells are the most potent and versatile antigen-presenting cells (APCs) capable of inducing protective adaptive immune responses and tolerance to self-antigens. In response to a variety of microbial and endogenous stimuli, resting DCs undergo a maturation process and migrate to the lymph node where they activate the adaptive immune response [9].

Chemokines control leukocyte migration and fulfill essential functions in homeostatic and inflammatory immune processes. CC chemokine ligand 1 (CCL1) is a CC chemokine mainly secreted by immune cells (e.g., Mo, activated macrophages and T cells, DCs and mast cells), dermal blood vasculature, and by melanocytes and Langerhans cells in the skin [10,11,12]. Human CCL1 interacts uniquely with the CC chemokine receptor 8 (CCR8). Early data on the expression of this receptor pointed to CCR8 being expressed by a number of T cell subsets, including Th1, Th2, T regulatory (Treg) cells, and thymocytes with natural Treg function, whereas expression and function of CCR8 in monocytes, DCs, and NK cells is still controversial [12,13]. The main function of the CCL1 interaction with CCR8 is the steady-state homing regulation of long-lived lymphocyte populations to skin tissue [10,14,15]. Nowadays, the CCL1/CCR8 axis is one of the least understood chemokine systems in spite of the fact that CCL1 was the first among a long succession of CC chemokines to be discovered [16,17]. Several functions have been attributed to CCL1 including the regulation of Treg and Th2 cells, as well as DC trafficking [10,18,19], IFN-γ production by CD8^+^ T cells [20], and allergic mucosal inflammation [10,21]. Inflammatory cytokines (*i.e.*, TNF-α and IL-1β) are known to induce CCL1 expression from cultured dermal endothelium [10]. DCs and their Mo precursors express moderate to large amounts of CCL1 mRNA upon activation with bacterial lipopolysaccharide (LPS) or peptidoglycan [10]. In addition, IL-4, IL-13, and IFN-γ can enhance CCL1 production from bronchial epithelial cells [22]. Lastly, in human Mo, CCL1 is induced by costimulation with FcγRII engagement and IL-1 or LPS [23,24], as well as by toll-like receptor (TLR)3, TLR4 and TLR8 ligands [25].

In this study, we report that bovine LF (bLF) up-modulates CCL1 expression in Mo-derived DCs (MoDCs) and their Mo precursors by distinct mechanisms involving the trans-membrane glycoprotein TLR2 and CD36. Overall, these cell-specific bLF-mediated effects may represent a strategy to elicit anti-inflammatory responses in specific body districts, where these cell types may be differently represented in physiopathological conditions.

## 2. Results

### 2.1. bLF Up-Modulates CCL1 Expression in Mo and MoDCs

**Figure 1:**
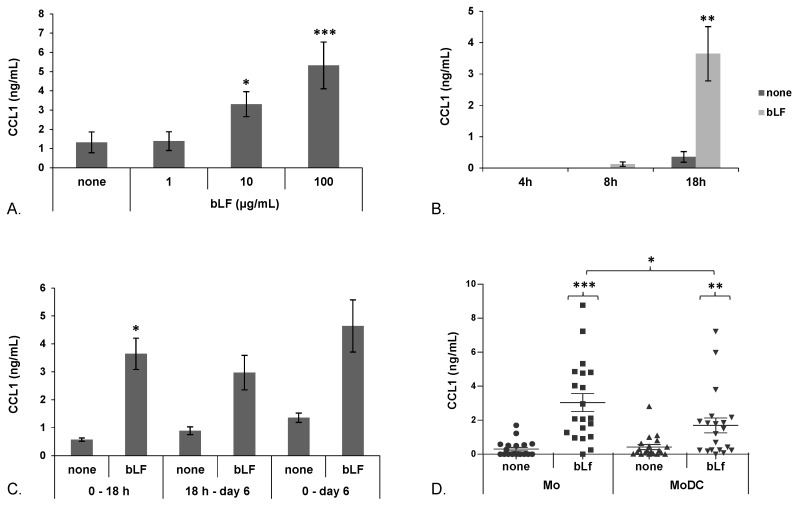
bLF-induced CCL1 secretion in Mo and MoDCs. Freshly isolated Mo were stimulated to differentiate to MoDCs in the presence of GM-CSF/IL4 and concomitantly treated with bLF or left untreated (none). (**A**) CCL1 content in culture supernatants of Mo stimulated with different concentrations of bLF for 18 h. Means ± SEM from six independent donors are shown; (**B**) Time-course analysis of CCL1 secretion. Mo were treated with 100 μg/mL of bLF for the indicated times or left untreated (none). Means ± SEM from eight (4 h), four (8 h) and 10 (18 h) independent experiments are shown; (**C**) Mo were stimulated to differentiate in the presence of GM-CSF/IL-4 and concomitantly treated with 100 μg/mL of bLF or left untreated for 18 h (0 to 18 h), then culture medium was replaced with fresh medium containing GM-CSF/IL-4 and cells were cultured for an additional five days (18 h to Day 6). Some cultures were treated with bLF soon after seeding, concomitantly to GM-CSF/IL-4, and culture supernatants were collected after six days (0 to Day 6). Data from four independent donors are shown; (**D**) Dot plot of CCL1 content in cell supernatants from Mo or MoDCs obtained from 20 independent donors. Cells were treated with 100 μg/mL bLF for 18 h or left untreated. Means ± SEM are indicated; (**A**) *p* values obtained by 1-way ANOVA (overall *p* < 0.0001) are shown; (**B**–**D**) *p* values obtained by Student *t* test for paired samples are shown. * *p* ≤ 0.05, ** *p* ≤ 0.01, *** *p* ≤ 0.001.

To get insights into bLF capacity to modulate cytokine/chemokine expression during the course of DC differentiation, Mo induced to differentiate toward DCs were treated with bLF at day 0 (Mo) or at day 5 (MoDCs) of culture and supernatants collected after 18 h. We found that Mo respond to bLF by up-modulating CCL1 secretion over basal levels. As shown in Figure 1A, bLF exposure of Mo soon after seeding influences CCL1 production in a concentration-dependent manner. Although no differences in the production of this chemokine were observed upon addition of 1 μg/mL of bLF with respect to the untreated control culture, CCL1 secretion was already observed with 10 μg/mL (3.3 fold increase *versus* control; *p* < 0.01; *n* = 6), and further increased at the concentration of 100 μg/mL of bLF (5.6 fold increase *versus* control; *p* < 0.01; *n* = 6). Low levels of CCL1 secretion were already detected at 8 h after bLF treatment but strongly increased at 18 h (Figure 1B). In addition, CCL1 was still detected in the culture supernatant at the end of the culture period (six days), when Mo treated with bLF have fully differentiated into MoDCs, even upon medium replacement 18 h post bLF treatment (Figure 1C), thus indicating that CCL1 secretion is not a transient event but continues in spite of stimulus removal. We have previously demonstrated that freshly isolated Mo, but not MoDCs, respond to bLF by secreting IL-6 [26]. Thus, we comparatively assessed the effect of a single treatment with bLF (100 μg/mL) in day 0 Mo or fully differentiated day 5 MoDCs on the production of this chemokine. As shown in Figure 1D, in spite of a clear-cut variability among donors in term of basal levels of CCL1 release and extent of response, bLF significantly up-modulated CCL1 secretion in both cell types. However, chemokine production was significantly higher in cultures stimulated at day 0 (Mo) with respect to those treated at day 5 (MoDCs).

**Figure 2:**
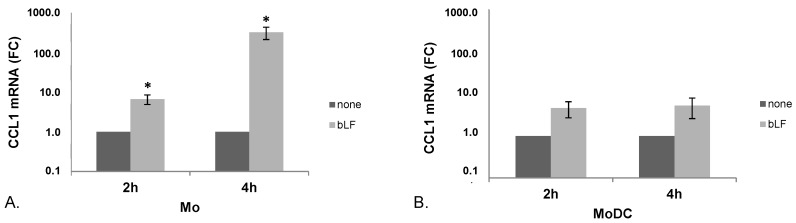
CCL1 mRNA accumulation in bLF-exposed Mo and MoDCs. CCL1 mRNA expression was analyzed by quantitative PCR and expressed as 2^−ΔΔCT^ values. Actin was used as reference gene, and the untreated sample as calibrator. Data are expressed as mean ± SEM of the fold changes (FC) with respect to untreated cells from seven (**A**, Mo) and four (**B**, MoDC) independent experiments. *p* values obtained by Student *t* test for paired samples are shown. * *p* ≤ 0.05.

To further establish whether bLF-stimulated CCL1 production was associated with an increased accumulation of the corresponding mRNA, RT-PCR experiments were performed at early time points after bLF treatment. As shown in Figure 2A, in day 0 Mo, bLF significantly enhanced CCL1 mRNA expression already at 2 h and more markedly at 4 h post-treatment. Although MoDCs exposed to bLF showed a trend towards a higher accumulation of CCL1 mRNA at both time points assessed (4.6 and 5.3 fold increase at 2 and 4 h post treatment, respectively), the differences did not reach statistical significance (Figure 2B).

### 2.2. bLF-Mediated Up-Modulation of CCL1 Secretion Requires Different Receptors in Mo and MoDCs

**Figure 3:**
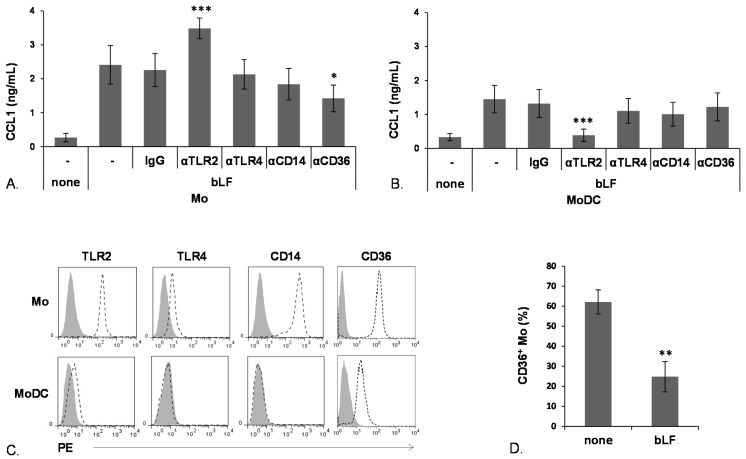
bLF up-modulation of CCL1 secretion requires different receptors in Mo and MoDCs. Mo (**A**) and MoDCs (**B**) were pretreated for 30 min with 5 μg/mL of the indicated mAbs, or left untreated (none), and then stimulated with bLF (100 μg/mL) for 18 h. Culture supernatants were collected and analyzed for CCL1 content by ELISA. Mean ± SEM from 11 (Mo) or 14 (MoDCs) independent experiments are shown. Statistical significance of the comparison between bLF stimulated cells pretreated with neutralizing Ab *versus* cells treated with control IgG isotype was calculated by one-way ANOVA (overall *p* < 0.0001); (**C**) Flow cytometry analysis of surface expression of the indicated receptors. Solid histograms represent control isotype; dotted lines the specific Ab. Data from one donor representative of four analyzed are shown; (**D**) Flow cytometry analysis of CD36 surface expression upon treatment of Mo with bLF (100 μg/mL) for 18 h. The percentage of CD36 positive cells is shown. Mean ± SEM from nine independent experiments are shown. *p* values obtained by Student *t* test for paired samples are shown. * *p* ≤ 0.05, ** *p* ≤ 0.01, *** *p* ≤ 0.001.

LF can interact, specifically or by virtue of its highly cationic nature, with a variety of cell determinants with different grade of specificity, including molecules involved in pathogen recognition such as DC-SIGN, CD14, TLR2 and TLR4 [4,5,27]. In this regard, we have previously demonstrated that engagement of TLR2, CD14 and, to a lesser extent, of TLR4 accounts for bLF-induced expression of IL-6 in Mo. Furthermore, we reported that cell-specific differences in bLF internalization likely account for the distinct response elicited by bLF in Mo *versus* MoDCs [26]. With the purpose of further investigating the mechanisms through which bLF promotes CCL1 expression in Mo and MoDCs, the effect of neutralizing antibodies specific for TLR4, TLR2 and their co-receptors CD14 and CD36 was evaluated. As shown in Figure 3, blocking CD36 significantly reduced the bLF-mediated CCL1 production in Mo (panel A) but not in MoDCs (panel B). Furthermore, significant inhibition of CCL1 release was not observed in both cell types upon blocking TLR4 or CD14 (Figure 3, panels A and B). Surprisingly, neutralization of TLR2 significantly enhanced the bLF-induced CCL1 release in Mo (Figure 3A), whereas it markedly inhibited the production of this chemokine in MoDCs (Figure 3B). To unravel possible correlations between the degree of receptor expression and CCL1 induction, the surface expression of CD36 and TLR2, and of CD14 and TLR4 as controls, was analyzed in Mo and MoDCs. As shown in Figure 3C, both cell types expressed TLR2 and CD36, although at different extent, while TLR4 expression was detected in Mo and barely in MoDCs. As expected, Mo but not MoDCs exhibited CD14 expression. CD36 has not been recognized yet as a receptor for LF although evidence for CD36-LF interaction has been previously provided [28]. Thus, to further investigate the role of CD36 in mediating bLF-induced activities in Mo, the effect of bLF exposure on the surface expression of this receptor was investigated. As shown in Figure 3D, treatment of Mo with bLF for 18 h resulted in a marked decrease of CD36 surface expression (from >60% to <30%), which suggests that receptor internalization occurs upon bLF engagement.

## 3. Discussion

LF is nowadays recognized as a first-line defense protein that plays a critical role in the regulation of immune response to infectious assault, trauma and injury, naturally bridging innate to adaptive immune functions. Although the mechanisms underlying the immunomodulatory effects of LF have not been fully elucidated, its capacity to modulate cytokine/chemokine production has been shown to contribute importantly to this function. The secretion of pro-inflammatory factors as well as of effector cytokines can be profoundly affected by LF either to increase or decrease their production, depending upon the type of insult recognized by the immune system in concert with local environmental conditions [4,5,6].

In this study, we report for the first time that bLF exposure of Mo and MoDCs results in a marked increase of CCL1 secretion. This effect appears to be significantly stronger in Mo with respect to MoDCs. Monocytes’ differentiation toward macrophages has been previously associated with a different capacity to spontaneously produce chemokines as well as to respond to external stimuli that modulate their secretion. In this regard, we have previously shown that CCL2 and CCL4 are constitutively expressed at high levels in human peripheral blood Mo, and their expression is further up-modulated during their differentiation into macrophages [29,30]. Likewise, CCL22 expression is first detected in Mo and reaches maximum levels in fully matured macrophages [31]. Differentiation of Mo toward macrophages also modulates the capacity to produce CC chemokine (*i.e.*, CCL2, CCL3, CCL4) in response to IFN-β [30] as well as to produce CCL1 in response to TLR engagement [25]. Conversely, little information is available on the influence of Mo differentiation toward DCs on the expression of chemokines. In this study, we show that MoDCs respond less to bLF, with respect to Mo, as lower levels of CCL1 are found upon induction. In keeping with this observation, we previously reported that Mo differentiation to MoDCs negatively correlates with the capacity to produce IL-6 in response to bLF [26]. In contrast to the transient onset of IL-6 production, CCL1 was found to be continuously produced during the course of differentiation as a consequence of the initial bLF stimulation at the stage of Mo. Overall, these observations suggest the existence of a certain degree of specificity in the bLF-mediated cytokine/chemokine induction, at least in part related to the differentiation stage, that may be relevant for the exploitation of LF function in specific body districts.

Herein, we also report that bLF-induced secretion of CCL1 is preceded by enhanced accumulation of CCL1 mRNA over the basal levels expressed by Mo and MoDCs. Although a clear-cut increase of this transcript was observed in both cell types upon bLF treatment, statistical significance was achieved in Mo but not in MoDCs, likely reflecting their lower capacity to secrete CCL1 in response to bLF (Figure 1D). The increased accumulation of CCL1 transcripts may be either due to an enhancement of the basal level of transcription or to a stabilization of CCL1 mRNA, both of which would result in the accumulation of this transcript. LF is known to be internalized and translocated into the nucleus in some cell types including Mo, where it may regulate the expression of target genes [26,32,33]. Furthermore, previous reports have documented that LF and its isoform delta-LF, regulate a number of genes via activation of signaling pathways or via binding to DNA [34,35]. Although further experiments are needed to precisely define the mechanism of action of bLF in the regulation of CCL1 mRNA expression, it cannot be ruled out that the more robust accumulation of CCL1 mRNA in Mo may rely on internalization and nuclear localization of bLF occurring in Mo but not in MoDCs.

In this study, we also report that bLF engages different receptors to trigger CCL1 secretion in Mo and MoDCs. Due to its cationic nature, LF binds with a different grade of specificity a variety of cellular determinants, including bacterial components, strongly anionic molecules, CD14, pathogen recognition receptors including *C*-type lectin receptors and some TLRs [27,36,37,38,39,40,41]. Although the relevance of these receptors in triggering bLF effects in human primary Mo and MoDCs is still poorly known, it is of interest that major differences in the expression of at least some of these receptors (e.g., CD14 and DC-SIGN) as well as in their involvement in specific LF effects have been reported in Mo with respect to MoDCs. In this regard, we have previously demonstrated that CD14 and its co-receptors TLR2 and TLR4, although not involved in bLF uptake by Mo, play a role in bLF-induced signaling leading to IL-6 expression. Since CD14 is barely or not expressed at all at the surface of MoDCs, and IL-6 is not induced by bLF in these cells, these results suggested that CD14 may represent an important determinant for the differential effects induced by bLF in the two cell types. However, the results achieved in this study rule out a possible involvement of CD14 in CCL1 induction, and introduce an additional level of complexity. Although CCL1 induction is independent from CD14 and TLR4, the differentiation stage influences, at least quantitatively, CCL1 expression, and relies on the engagement of two different receptors, CD36 in Mo and TLR2 in MoDCs. Interestingly, receptors that are engaged by LF to induce specific effects in myeloid immune cells, take part in receptor complexes that play a crucial role in governing inflammation pathways such, as TLR4 [42] and TLR2 [43]. Likewise, CD36 facilitates TLR2 recognition and, together with CD14, is required for TLR2 response to lipoteichoic acid [44,45,46]. On the basis of these observations and of our own results, we speculate that LF effects may rely on receptor complexes, differently assembled depending on the cell type and differentiation stage, that may positively or negatively interact to mediate LF-induced effects. The apparent paradoxical effect of TLR2 blocking in Mo, up-modulating CCL1 secretion rather than inhibiting as in MoDCs, can be likely explained by the complex interplay of several actors diversely assembled/triggered by bLF at early and late differentiation stages.

We have previously reported that bLF skews Mo differentiation into DCs with impaired capacity to undergo activation and to promote Th1 responses, likely representing a strategy to block excessive DC activation upon TLR-induced inflammation [26]. However, our attempts to link CCL1 to the generation of DCs with distinct properties have failed (data not shown), and the precise role of this chemokine in the bLF immunomodulatory effects still remains to be elucidated.

**Figure 4:**
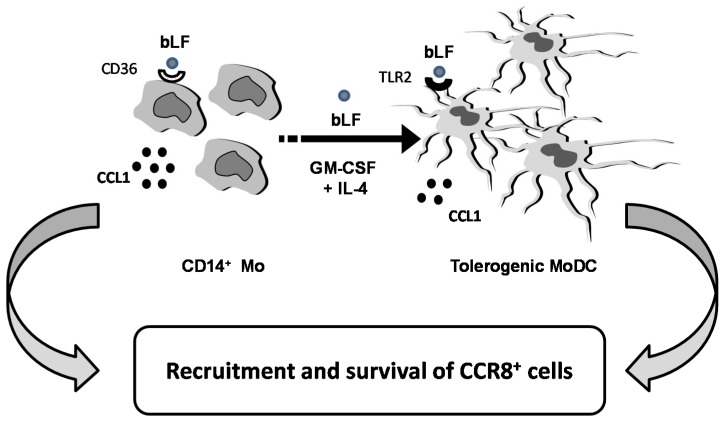
Schematic model of the proposed role of bLF-induced CCL1.

CCL1 plays a role not only in inflammation but also in apoptosis, angiogenesis and tumor biology. Overall, the results of this study add further evidence for a critical role of bLF in directing host immune function. As schematically depicted in Figure 4, Mo differentiating into DCs in the presence of bLF acquire a tolerogenic phenotype and produce, throughout their differentiation process, CCL1. We speculate that this chemokine may contribute to the anti-inflammatory activity of bLF by recruiting CCR8^+^ cells (*i.e.*, Treg and Th2 cells) and, by virtue of its protective effect against apoptosis, to ensure the survival of the recruited cells, thus dampening inflammation.

Taken together, these findings shed some light on the mechanisms underlying bLF anti-inflammatory activity, highlighting its potential as a key component in the regulation of the inflammatory response.

## 4. Experimental Section

### 4.1. Ethics Statements

Healthy donor buffy coats were obtained from Centro Trasfusionale, University of Rome Sapienza. Buffy coats were not obtained specifically for this study. Informed consent has not been asked because data were analyzed anonymously. Data from healthy donors have been treated by Centro Trasfusionale according to the Italian law on personal data management, “Codice in materia di protezione dei dati personali” (Testo Unico D.L., 30 June 2003, No. 196).

### 4.2. Reagents

Highly purified bLF in lyophilized form was kindly provided by Morinaga Milk Industries Co., Ltd., (Tokyo, Japan). bLf was checked for purity by Sodium Dodecyl Sulphate-Polyacrilamide Gel Electrophoresis (SDS-PAGE) (Biorad, Segrate, Italy) and found to be contaminant-free, with a single band displayed at 80 kDa upon staining with silver nitrate. The iron saturation of bLf was about 25%, as determined by optical spectroscopy (PerkinElmer, Monza, Italy). Before biological assays, bLf preparations were sterilized by filtration using 0.2 μm filter (Millipore Corp., Bedford, MA, USA) at low protein retention [47]. bLf contains a minimal amount of LPS, *i.e.*, 0.7 EU/mg protein, corresponding to 100 pg/mL free LPS, by conventional Limulus Amebocyte Assay (Charles River Endosafe, Charleston, SC, USA; detection limit: 0.125 endotoxin units/mL). However, at this LPS concentration CCL1 secretion was not affected, and neither anti-TLR4 mAb influenced bLF capacity to induce CCL1 secretion (data not shown and Figure 3). Recombinant human GM-CSF and IL-4 were a generous gift from Schering-Plough (Dardilly, France). Neutralizing monoclonal antibodies (mAbs) against TLR4 (clone 15C1) [48] and TLR2 (clone T 2.5) [49] were kindly provided by Greg Elson, anti-CD14 (clone 134620) and IgG1k isotype control Ab (clone 11711) were purchased from R&D, anti-CD36 (clone FA6-152) was provided by GENETEX Inc. (Irvine, CA, USA) PE-conjugated anti-CD14 (clone MФP9), anti-CD36 (clone CB38) and the corresponding isotype Ab were obtained from BD Biosciences (Milan, Italy). PE-conjugated anti-TLR4 (clone HTA125), anti-TLR2 (T2.5) and control Abs were from eBioscience.

### 4.3. Cell Isolation and Culture

Peripheral blood mononuclear cells (PBMCs) were isolated from healthy donors buffy-coats by Ficoll-Paque density centrifugation. CD14^+^ monocyte population was purified by positive immunomagnetic bead selection (MACS monocyte isolation kit II from Miltenyi Biotec, Calderara di Reno, Italy), according to the manufacturer’s instructions as previously described [50]. Freshly isolated Mo were resuspended in RPMI 1640 medium Life Technologies, Monza, Italy) supplemented with 2 mM *L*-glutamine, 2 mM penicillin/streptomycin and 10% fetalbovineserum (FBS) (Hyclone), seededat 1 × 10^6^ cells/mL and treated with bLF in the presence of GM-CSF (50 ng/mL) and IL-4 (500 U/mL) to generate immature MoDCs [51]. GM-CSF and IL-4 were replenished at days 2 and 5 of culture. Cells were cultured at 37 °C, in a 5% CO_2_ and 95% H_2_O atmosphere.

### 4.4. CCL1 mRNA and Protein Detection

Total mRNA was extracted and processed for RT-PCR experiments as elsewhere indicated [52]. Validated PCR primers and TaqMan MGB probe (6FAM-labeled) for CCL1 were used (Hs00171072 m1; Applied Biosystems, Monza, Italy). As endogenous control, primers with TaqMan probe for the human β actin were used (Hs99999903 m1; Applied Biosystems). CCL1 concentration was measured by homemade ELISA, following the manufacturer’s instructions (sensitivity 15.6 pg/mL, R&D System, Minneapolis, MN, USA).

### 4.5. Flow Cytometry

Cells were pre-incubated with PBS containing 10% human AB serum for 30 min on ice to block unspecific Ig binding, then incubated with the specific or control Ab, respectively. After 30 min of incubation on ice, cells were washed and fixed in 1% formaldehyde. Samples were acquired with a FACS Calibur flow cytometer by using Cell Quest (Becton Dickinson, Milan, Italy) and data analyzed by FlowJo software (Tree Star, Inc., Ashland, OR, USA).

### 4.6. Statistical Analysis

Statistical comparison between different experimental conditions was determined by the Student’s *t* test (paired, two-tailed) by using SPSS and Graphpad software Inc. (La Jolla, CA, USA). Where not specifically indicated by graph parenthesis, statistical significance was calculated with respect to the untreated cells. When different treatments were performed, data were analyzed by one-way ANOVA for repeated measures. The Tukey’s post-test was applied for multiple comparisons. *p* values ≤ 0.05 were considered to reflect statistical significance; * *p* ≤ 0.05, ** *p* ≤ 0.01, *** *p* ≤ 0.001.
